# Synthesis and Radioprotective Activity of New Organosilicon and Germanium Compounds

**DOI:** 10.1155/MBD.1998.139

**Published:** 1998

**Authors:** Ghassoub Rima, Jacques Satgé, Rodolphe Dagiral, Claude Lion, Marc Fatome, Vincent Roman, Jean-Denis Laval

**Affiliations:** 1 Laboratoire d′Hétérochimie Fondamentale et Appliquée UPRES-A 5069 du CNRS Université Paul Sabatier, 118 route de Narbonne Toulouse Cedex 31062 France; 2 Institut de Topologie et de Dynamique des Systèmes de I'Université de Paris VII Associé au CNRS rue Guy de la Brosse Paris 75005 France; 3 Unité de Radioprotection Centre de Recherches du Service de Santé des Armées, 24 avenue des Maquis du Grésivaudan La Tronche Cedex 38702 France

## Abstract

Silathiazolidine and metalladithioacetals (M = Si, Ge) have been prepared by the interaction of dialkyldichloro- or bis(diethylamino)dialkylsilanes and -germanes with 3-[N-(2- thioethyl)]amino-propanamide (WR-2529) and [1-thioethyl-2-(1-naphtylmethyl)]-2- imidazoline. The study of these compounds in the field of chemical radioprotection has shown a notable decrease in the toxicity and a rather large increase in the radioprotective activity of these new derivatives in comparison with the starting organic compounds.


### SYNTHESIS AND RADIOPROTECTIVE ACTIVITY OF NEW ORGANOSILICON AND GERMANIUM COMPOUNDS

Ghassoub Rima[1], Jacques Satgé*[1], Rodolphe Dagiral[1],

Claude Lion[2], Marc Fatome[3], Vincent Roman[3] and Jean-Denis Laval[3].

1 Laboratoire d'Hétérochimie Fondamentale et Appliquée, UPRES-A 5069 du CNRS, Université Paul Sabatier, 118, route de Narbonne, 31062 Toulouse Cedex, France.

2 Institut de Topologie et de Dynamique des Systèmes de l'Université de Paris VII, Associé au CNRS, 1 rue Guy de la Brosse, 75005 Paris, France.

3 Unité de Radioprotection, Centre de Recherches du Service de Santé des Armées, 24, avenue des Maquis du Grésivaudan, 38702 La Tronche Cedex, France.

**Abstract**

Silathiazolidine and metalladithioacetals (M = Si, Ge) have been prepared by the interaction of dialkyldichloro- or bis(diethylamino)dialkylsilanes and -germanes with 3-[N-(2-thioethyl)]amino-propanamide (WR-2529) and [1-thioethyl-2-(1-naphtylmethyl)]-2-imidazoline. The study of these compounds in the field of chemical radioprotection has shown a notable decrease in the toxicity and a rather large increase in the radioprotective activity of these new derivatives in comparison with the starting organic compounds.

**Introduction**

Current interest in the radioprotective activity of several classes of organosilicon and organogermanium derivatives is attested by a growing number of reported syntheses in this area [1-10]. This report concerns the synthesis, toxicity and study of the biological activity of some new silathiazolidine, sila- and germadithioacetals (see scheme).

**Materials and methods**

All manipulations were carried out under dry nitrogen. Solvents were freshly distilled from sodium/benzophenone before use. IR spectra were recorded on a Perkin-Elmer 1600FT-IR spectrophotometer. $^{1}H$ and $^{13}C$ NMR spectra were recorded on Bruker's AC 80 (80.13 MHz) and AC 200 (50.32 MHz) spectrometers; the multiplicity of the $^{13}C$ NMR signals was determined by the APT technique and quoted (-) for $CH_3$ and CH, (+) for $CH_2$ and ($C_{quat}$) for quaternary carbon atoms. Mass spectra under electron impact (EI) conditions at 70 and 30 eV were recorded on a Hewlett-Packard 5989A spectrometer. Elemental analyses (C, H, N) were performed at the Laboratoire de Microanalyse de l'Ecole Nationale Supérieure de Chimie de Toulouse.

Scheme

**Silathiazolidine**

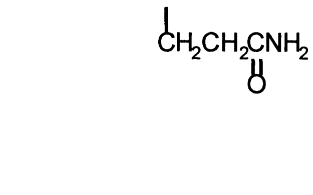

1

**[1-Thioethyl-2-(1-naphthylmethyl)]-2-imidazoline**

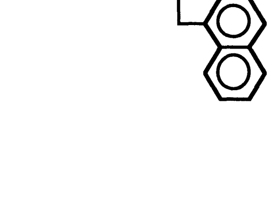

8

**Sila- and germadithioacetals**

$R_2M(SCH_2CH_2R')_2$

**M = Si**

R' = $-NHCH_2CH_2C(O)NH_2$

R = $i$-$C_5H_{11}$ **2**

R = $n$-$C_6H_{13}$ **3**

R' = 

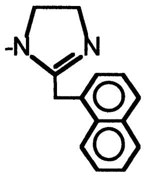

R = $i$-$C_5H_{11}$ **4**

R = $n$-$C_6H_{13}$ **5**

**M = Ge**

R' = 

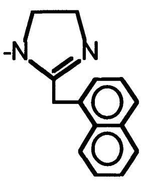

R = $i$-$C_5H_{11}$ **6**

R = $n$-$C_6H_{13}$ **7**

**Silathiazolidine 1**

This compound was prepared by two methods: A and B

**Method A**

Di-$n$-hexyldichlorosilane (3.91 g, 14.51 mmol) in 50 ml of THF was added dropwise to a stirred mixture of 3-[N-(2-thioethyl)]aminopropanamide [11] (2.15 g, 14.51 mmol) and freshly distilled triethylamine (3.23 g, 31.92 mmol) in 70 ml of THF. The reaction mixture was refluxed for 2 h with stirring. After cooling, the mixture was filtered under nitrogen to remove the precipitate $Et_3N.HCl$. Removal of volatiles (under reduced pressure) from the filtrate, the residue was extracted by 40 ml of dry pentane. Filtration and concentration, afforded **1** (3.88 g, 78 %).

**Method B**

To a stirred mixture of 3-[N-(2-thioethyl)]aminopropanamide [11] (1.60 g, 10.80 mmol) in 50 ml of THF was added dropwise, a solution of bis(diethylamino)di-$n$-hexylsilane (3.70 g, 10.80 mmol) in 50 ml of THF. The mixture was refluxed under nitrogen for 3 h. The volatile material was removed *in vacuo* to afford the compound **1** (3.57 g, 96 %).

**Sila- and germadithioacetals 2-7**

These compouds were also synthesized by two methods: C and D

**Method C**

Diisoamyldichlorosilane (1.82 g, 7.55 mmol) in 30 ml of THF was added dropwise to a stirred mixture of 3-[N-(2-thioethyl)]aminopropanamide (2.24 g, 15.11 mmol) and freshly distilled triethylamine (1.68 g, 16.62 mmol) in 50 ml of THF. After refluxing for 3 h, the resulting mixture was cooled down to room temperature, filtered and evaporated under vacuum. The residue was extracted in 30 ml of dry pentane. Filtration, followed by removal of the solvent under vacuum gave **2** (1.99 g, 57 %).

**Method D**

Bis(diethylamino)di-$n$-hexylsilane (2.00 g, 5.84 mmol) in 40 ml of THF was added dropwise with stirring to a suspension of $HSCH_2CH_2NHCH_2CH_2C(O)NH_2$ (1.73 g, 11.67 mmol) in 70 ml of THF. The mixture was refluxed under nitrogen for 3 h. After cooling down to room temperature, the volatiles were removed under vacuum to afford **3** (2.02 g, 70 %).

Compounds **4-7** were prepared analogously from the appropriate dialkyldichlorometallane or bis(diethylamino)dialkylmetallane and 3-[N-(2-thioethyl)]aminopropanamide or [1-thioethyl-2-(1-naphtylmethyl)]-2-imidazoline.

**Table I - Physicochemical data and analyses of derivatives 1-8**

| Compound | Method of synthesis | Yield (%) | Physical Properties and Elemental Analyses |
|---|---|---|---|
| **1** | A or B | 96 | $^1$H NMR ($CDCl_3$; δ, ppm): 0.84 (t, 6H, J = 5.56 Hz); 0.91 (t, 4H, J = 7.55 Hz); 1.08-1.52 (m, 16H); 2.29-3.02 (m, 8H). $^{13}$C NMR ($CDCl_3$; δ, ppm): 13.41 (+); 14.09 (-); 14.51 (+); 22.35 (+); 22.55 (+); 31.49 (+); 32.76 (+); 35.40 (+); 44.84 (+); 51.73 (+); 175.25 (C=O). I.R. ($CCl_4$; $cm^{-1}$): $\nu_{C=O}$ = 1674; $\nu_{NH_2}$ = 3182, 3318. Mass spectrum: m/z = 273 $[M -71]^+$. Analysis ($C_{17}H_{36}N_2OSSi$) Calcd %: C, 59.25; H, 10.53; N, 8.13. Found %: C, 59.16; H, 10.51; N, 8.06. |
| **2** | C or D | 57 | $^1$H NMR ($CDCl_3$; δ, ppm): 0.85 (d, 12H, J = 5.8 Hz); 0.97-1.56 (m, 10H); 2.31-2.98 (m, 16H). $^{13}$C NMR ($CDCl_3$; δ, ppm): 10.95 (+); 21.99 (-); 24.76 (+); 30.49 (-); 31.19 (+); 35.42 (+); 44.84 (+); 51.71 (+); 170.76 (C=O). I.R. ($CCl_4$; $cm^{-1}$): $\nu_{C=O}$ = 1727; $\nu_{NH_2}$ = 3202, 3344. Mass spectrum: m/z = 317 $[M - 147]^+$. Analysis ($C_{20}H_{44}N_4O_2S_2Si$) Calcd %: C, 51.68; H, 9.54; N, 12.05. Found %: C, 51.58; H, 9.71; N, 11.95. |
| **3** | C or D | 70 | $^1$H NMR ($CDCl_3$; δ, ppm): 0.85 (t, 6H, J = 5.7 Hz); 0.92 (t, 4H, J = 7.55 Hz); 1.07-1.58 (m, 16H); 2.35-3.35 (m, 16H). $^{13}$C NMR ($CDCl_3$; δ, ppm): 14.17 (-); 15.67 (+); 22.65 (+); 23.02 (+); 29.45 (+); 31.61 (+); 32.99 (+); 37.45 (+); 43.82 (+); 52.07 (+); 170.18 (C=O). I.R. ($CCl_4$; $cm^{-1}$): $\nu_{C=O}$ = 1664; $\nu_{NH_2}$ = 3189, 3259. Mass spectrum: m/z = 345 $[M – 147]^+$. Analysis ($C_{22}H_{48}N_4O_2S_2Si$) Calcd %: C, 53.61; H, 9.81; N, 11.37. Found %: C, 53.31; H, 9.92; N, 11.16. |
| **4** | C or D | 81 | $^1$H NMR ($CDCl_3$; δ, ppm): 0.84 (d, 12H, J = 5.7 Hz); 0.98-1.59 (m, 10H); 2.38-2.90 (m, 4H); 3.06-3.76 (m, 12H); 4.06 (s, 4H); 7.33-7.53 (m, 8H); 7.67-7.89 (m, 4H); 8.01-8.17 (m, 2H). $^{13}$C NMR ($CDCl_3$; δ, ppm): 12.76 (+); 22.16 (-); 24.61 (+); 30.81 (-); 32.23 (+); 32.37 (+); 50.24 (+); 50.37 (+); 52.43 (+); 123.92 (-); 125.51 (-); 125.74 (-); 126.30 (-); 126.43 (-); 127.65 (-); 128.75 (-); 131.85 ($C_{quat}$); 132.02 ($C_{quat}$); 133.88 ($C_{quat}$); 165.22 ($C_{quat}$). Mass spectrum: m/z = 439 $[M - 269]^+$. Analysis ($C_{42}H_{56}N_4S_2Si$): Calcd %: C, 71.14; H, 7.96; N, 7.90. Found %: C, 70.97; H, 7,84; N, 7.81. |
| **5** | C or D | 94 | $^1$H NMR ($CDCl_3$; δ, ppm): 0.86 (t, 6H, J = 5.7 Hz); 1.14-1.54 (m, 20H); 2.28-2.90 (m, 4H); 3.08-3.78 (m, 12H); 4.08 (s, 4H); 7.33-7.42 (m, 8H); 7.68-7.90 (m, 4H); 8.06-8.17 (m, 2H). $^{13}$C NMR ($CDCl_3$; δ, ppm): 14.15 (-); 14.73 (+); 16.40 (+); 22.62 (+); 23.43 (+); 31.41 (+); 31.50 (+); 32.41 (+); 50.26 (+); 50.38 (+); 52.50 (+); 122.44 (-); 124.68 (-); 124.99 (-); 126.31 (-); 126.43 (-); 127.76 (-); 128.84 (-); 131.91 ($C_{quat}$); 132.06 ($C_{quat}$); 133.88 ($C_{quat}$); 165.19 ($C_{quat}$). Mass spectrum: m/z = 651 $[M - 85]^+$. Analysis ($C_{44}H_{60}N_4S_2Si$) Calcd %: C, 71.69; H, 8.20; N, 6.60. Found %: C, 71.62; H, 8.13; N, 6.67. |
| **6** | C or D | 98 | $^1$H NMR ($CDCl_3$; δ, ppm): 0.88 (d, 12H, J = 5.4 Hz); 1.09-1.42 (m, 10H); 2.34-2.74 (m, 4H); 3.07-3.76 (m, 12H); 4.05 (s, 4H); 7.31-7.56 (m, 8H); 7.69-7.88 (m, 4H); 8.07-8.18 (m, 2H). $^{13}$C NMR ($CDCl_3$; δ, ppm): 17.19 (+); 18.28 (+); 22.08 (-); 30.50 (-); 32.22 (+); 33.41 (+); 50.22 (+); 50.40 (+); 52.59 (+); 123.71 (-); 125.51 (-); 125.78 (-); 126.51 (-); 126.59 (-); 127.66 (-); 128.77 (-); 132.05 ($C_{quat}$); 132.43 ($C_{quat}$); 133.88 ($C_{quat}$); 165.07 ($C_{quat}$). Mass spectrum: m/z = 485 $[M - 269]^+$. Analysis ($C_{42}H_{56}N_4S_2Ge$) Calcd %: C, 66.93; H, 7.49; N, 7.43. Found %: C, 66.78; H, 7,32; N, 7.45. |

| | | | |
|---|---|---|---|
| **7** | C or D | 98 | $^1$H NMR ($CDCl_3$; δ, ppm): 0.87 (t, 6H, J = 5.6 Hz); 1.09-1.54 (m, 20H); 2.35-2.74 (m, 4H); 3.03-3.77 (m, 12H); 4.05 (s, 4H); 7.19-7.49 (m, 8H); 7.67-7.92 (m, 4H); 8.16-8.33 (m, 2H). $^{13}$C NMR ($CDCl_3$; δ, ppm): 14.11 (-); 19.85 (+); 22.54 (+); 23.36 (+); 24.64 (+); 31.15 (+); 31.57 (+); 32.35 (+); 50.25 (+); 50.44 (+); 52.71 (+); 123.72 (-); 125.52 (-); 125.80 (-); 126.35 (-); 126.56 (-); 127.62 (-); 128.75 (-); 132.10 ($C_{quat}$); 132.29 ($C_{quat}$); 133.89 ($C_{quat}$); 165.03 ($C_{quat}$). Mass spectrum: m/z = 697 $[M - 85]^+$. Analysis ($C_{44}H_{60}N_4S_2Ge$) Calcd %: C, 67.61; H, 7.74; N, 7.17. Found %: C, 67.35; H, 7.66; N, 7.54. |
| **8** | | 74 | $^1$H NMR ($CDCl_3$; δ, ppm): 2.45 (s, 1H); 2.56-2.85 ([illegible] 2H); 3.07-3.93 (m, 6H); 4.04 (s, 2H); 7.34-7.54 (m, 4H); 7.66-7.89 (m, 2H); 8.04-8.19 (m, 1H). $^{13}$C [illegible] ($CDCl_3$; δ, ppm): 23.44 (+); 32.31 (+); 50.15 (+); 50.31 (+); 52.60 (+); 123.68 (-); 125.54 (-); 125.85 (-); 126.45 (-); 126.52 (-); 127.77 (-); 128.86 (-); 132.02 ($C_{quat}$); 132.39 ($C_{quat}$); 133.88 ($C_{quat}$); 165.25 ($C_{quat}$). I.R. ($cm^{-1}$): $\nu_{SH}$ = 2538. Mass spectrum: m/z = 269 $[M - 1]^+$. Analysis ($C_{16}H_{18}N_2S$) Calcd %: C, 71.07; H, 6.71; N, 10.36. Found %: C, 70.86; H, 6.77; N, 9.99. |

**[1-Thioethyl-2-(1-naphtylmethyl)]-2-imidazoline 8**

A solution of 2-(1-naphtylmethyl)-2-imidazoline[#] (5.29 g, 25.16 mmol) in 60 ml of dry toluene was mixed with a solution of ethylene sulfide (1.59 g, 26.45 mmol) (Aldrich-Chemical) in 40 ml of dry toluene (sealed tube, argon flushed). The reaction mixture was then heated (110°C oven) for 15 h. After cooling 100 ml of cold diethyl ether was added with stirring to reaction mixture, and filtration to remove small amount of polyethylene sulfide. The solvent was removed under reduced pressure to give a yellow pasty product **8** (4.78 g, 74 %).

[#]To a solution of 2-(1-naphthylmethyl)-2-imidazoline hydrochloride (Aldrich-Chemical) (30g, 121.58 mmol) in 30 ml of water was added with stirring a solution of NaOH 14 % (34.73 g). After extraction with toluene (500 ml), the organic layer dried on $Na_2SO_4$. Removal of solvent *in vacuo* and crystallization from THF/diethyl ether (1/9, 400 ml) gave 2-(1-naphtylmethyl)-2-imidazoline in form of white crystals (21.31 g, 87 %).

Physicochemical data of derivatives **1-8** are reported in Table I

**Table II - Radioprotective activity of 1-8 compounds**

| Compound | $LD_{50}$: mg.kg$^{-1}$ (mmol) | Injected dose mg.kg$^{-1}$ | Irradiation Gy (t, min)[a] | Survival rate % | DRF[b] |
|---|---|---|---|---|---|
| **1** | > 1500 (4.35) | 1000<br>1000 | 8 (15)<br>8 (90) | 70<br>30 | 1.2 |
| **2** | > 800 (1.72) | 600 | 7.5 (15) | 20 | - |
| **3** | > 1500 (3.04) | 750 | 8 (15) | 60 | - |
| **4** | ~100 (0.14) | 50<br>50<br>12.5<br>50 | 7.75 (15)<br>7.75 (90)<br>7.75 (15)<br>9.75 (15) | 100<br>80<br>60<br>10 | 1.4 |
| **5** | ~100 (0.13) | 50<br>50<br>12.5 | 7.75 (15)<br>7.75 (90)<br>7.75 (15) | 100<br>100<br>50 | 1.5<br><br>1.2 |
| **6** | ~80 (0.11) | 50<br>50<br>50<br>12.5<br>50<br>50<br>50 | 7.75 (15)<br>7.75 (90)<br>7.75 (180)<br>7.75 (15)<br>9.75 (15)<br>9.75 (90)<br>11.75 (15) | 100<br>100<br>90<br>80<br>70<br>0<br>30 | 1.7<br><br><br><br><br>1.25 |
| **7** | ~150 (0.19) | 75<br>75<br>18.75<br>75 | 7.75 (15)<br>7.75 (90)<br>7.75 (15)<br>9.75 (15) | 90<br>100<br>70<br>10 | 1.4 |
| **8** | 35 (0.13) | 17.5<br>4.38 | 8 (15)<br>8 (15) | 80<br>47 | 1.1 |

a: t = time between administration of compound and irradiation.

b: dose reduction factor = ($LD_{50(30\ days)}$ treated/$LD_{50(30\ days)}$ untreated).

**Pharmacology: evaluation of radioprotection**

Male CD1 mice (Charles River, France), 25 g body weight, were used. Compounds were injected intraperitoneally 15, 90 or 180 min before irradiation. The irradiation dose was $LD_{100}$/30 days for untreated mice (7.5, 7.75 or 8 Gy, according to the irradiation date) or a 2

Gy greater dose. The injected dose of compound was equal to, three-quater, two-third, one-half or one eighth of the $LD_{50}$ value which had been determined previously. The radioprotective effect was evaluated by the Dose Reduction Factor (DRF), which is the ratio between the $LD_{50}/30$ days of treated mice and that of control mice (between 6.5 and 6.75 Gy, according to the date).

Irradiation was applied using a cobalt-60 source at the dose rate of 0.3-0.4 $Gy.min^{-1}$ according to the date. During irradiation, animals were placed in a Plexiglass box with 30 cells in a homogeneous field, 28.5 x 28.5 cm in area. Dosimetry was checked with an ionisation chamber dosimeter. The different $LD_{50}$ values were determined by probit analysis.

**Results and discussion**

**Silathiazolidine**

Silathiazolidine has been prepared according to two methods of heterocyclisation already described in the litterature [1, 12, 13].

**Method A**

The action of di-*n*-hexyldichlorosilane, in stoichiometric amounts, on 3-[N-(2-thioethyl)]aminopropanamide in refluxing anhydrous THF in the presence of freshly distilled triethylamine gave by a cyclisation reaction, with elimination of hydrochloric acid from Si-Cl and NH groups [13], the corresponding product, Scheme 1:

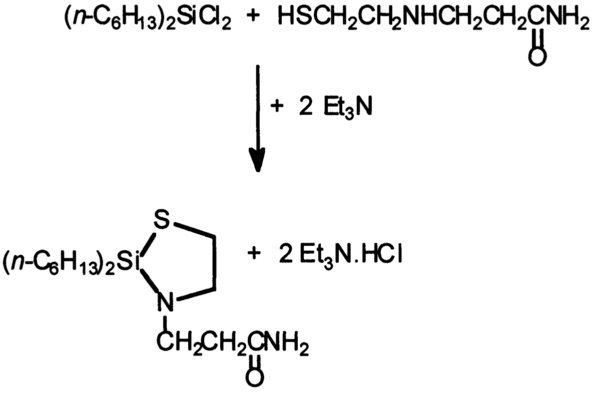

**Scheme 1**

**Method B**

Treatment of bis(diethylamino)-di-n-hexylsilane, in stoichiometric amounts, with 3-[N-(2-thioethyl)]aminopropanamide in anhydrous THF resulted in the cleavage of Si-N bonds by the N-H (a transamination reaction) and S-H groups [1,13,15] forming the corresponding silathiazolidine, Scheme 2:

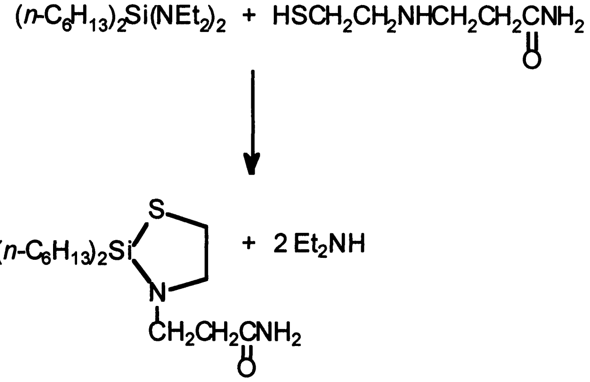

**Scheme 2**

**Sila- and germadithioacetals**

These compounds were also synthesized by two methods, C and D.

**Method C**

The reaction of dialkyldichlorosilanes and –germanes with two equivalents of 3-[N-(2-thio-ethyl)]aminopropanamide or [1-thioethyl-2-(1-naphtylmethyl)]-2-imidazoline in the presence of tri-ethylamine in refluxing anhydrous THF leads to the acyclic derivatives, Scheme 3:

$$R_2MCl_2 + 2\,R'CH_2CH_2SH \xrightarrow{+\ 2\,Et_3N} R_2M(SCH_2CH_2R')_2 + 2\,Et_3N.HCl$$

**Scheme 3**

**Method D**

The action of two equivalents of 3-[N-(2-thioethyl)]aminopropanamide or [1-thioethyl-2-(1-naphtylmethyl)]-2-imidazoline with bis(diethylamino)dialkylsilanes and -germanes in anhydrous THF, a cleavage reaction of M-N bonds by the N-H (a transamination reaction) and S-H groups [1, 13, 15] leads to the formation of the desired products, Scheme 4:

$$R_2M(NEt_2)_2 + 2\,R'CH_2CH_2SH \longrightarrow R_2M(SCH_2CH_2R')_2 + 2\,Et_2NH$$

**Scheme 4**

**Synthesis of [1-thioethyl-2-(1-naphtylmethyl)]-2-imidazoline**

This compound was obtained by the reaction of 2-(1-naphtylmethyl)-2-imidazoline with ethy-lene sulfide in a sealed tube at 110°C in anhydrous toluene (i.e. by a cleavage of the C-S bond by the N-H group) [16], Scheme 5:

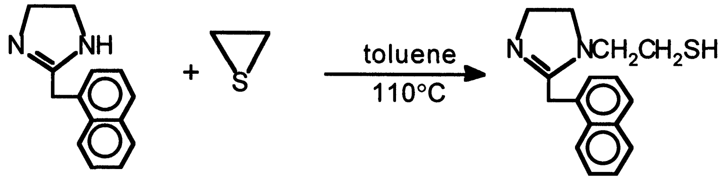

**Scheme 5**

**Conclusions**

The experimental evaluation of toxicity and radioprotective activity of metalladithioacetals and silathiazolidine **1-7** in the mice is presented in Table II.

Compounds **1** and **3** showed a lower toxicity ($LD_{50}$ > 1500 mg.kg$^{-1}$) compared with the starting organic derivative $HSCH_2CH_2NHCH_2CH_2C(O)NH_2$ (WR-2529) $LD_{50}$ = 700 mg.kg$^{-1}$ [10].

With the other sila- and germadithioacetals, derivatives **4-7**, we have observed a low decrease of the toxicity but a good radioprotective activity by intraperitoneal administration in mice. For example:

- derivative **4**: at $LD_{50}/2$ ~ 50 mg.kg$^{-1}$ this product protects 100 % and 80 % of mice when **4** was injected 15 and 90 minutes before irradiation.
- derivative **5**: at $LD_{50}/2$ ~ 50 mg.kg$^{-1}$ this product protects 100 % of mice when **5** was injected 15 and 90 minutes before irradiation. In the example shown, 50 % survival was also observed at $LD_{50}/8$.

Concerning the derivatives **6** and **7** we have noted a low toxicity and a notable increase of radioprotective activity:

- derivative **6**: at $LD_{50}/1.6$ ~ 50 mg.kg$^{-1}$ this product protects 100 % of mice when **6** was injected 15 and 90 minutes before irradiation, 90 % survival when **6** was injected 180 minutes before irradiation. At $LD_{50}/6.4$ there was still 80 % survival and at $LD_{50}/1.6$, 70 and 30 % survival at dose of 9.75 and 11.75 Gy.
- derivative **7**: at $LD_{50}/2$ ~ 75 mg.kg$^{-1}$ this product protects 90 and 100 % of mice when **7** was injected 15 and 90 minutes before irradiation. At $LD_{50}/8$, 70 % survival when **7** was injected 15 minutes before irradiation.

Chemical radioprotective study of silathiazolidine and metalladithioacetals showed a low toxicity and more potent protection than $HSCH_2CH_2NHCH_2CH_2C(O)NH_2$ (WR-2529) or derivative **8**.

The results reported in this paper confirm the positive contribution of silicon and germanium in this field in agreement with previous works [1-10] and the interesting biological activity of organosilicon and organogermanium compounds in different fields [17-25].

**Acknowledgments**

The authors are indebted to Direction générale de l'Armement (D.G.A.), Département de Chimie-Pharmacologie, Ministère de la Défense Nationale, France for their financial support and interest in this research.

**References**

1. J. Satgé, A. Cazes, M. Bouchaut, M. Fatome, H. Sentenac-Roumanou and C. Lion, *Eur. J. Med.Chem.*, **17**(1982)433.
2. M. Fatome, H. Sentenac-Roumanou, C. Lion, J. Satgé, M. Fourtinon and G. Rima, *Eur. J. Med.Chem.*, **19**(1984)119.
3. M. Fatome, H. Sentenac-Roumanou, C. Lion, J. Satgé and G. Rima, *Eur. J. Med. Chem.*, **23**(1988)257.
4. J. Satgé, G. Rima, M. Fatome, H. Sentenac-Roumanou and C. Lion, *Eur. J. Med. Chem.*, **24**(1989)48.
5. G. Rima, J. Satgé, C. Lion, H. Sentenac-Roumanou and D. Guyot, Synth. *React. Inorg. Met.-Org. Chem.*, **19**(1989)787.
6. G. Rima, J. Satgé, M. Fatome, J. D. Laval, H. Sentenac-Roumanou, C. Lion and M. Lazraq, *Eur. J. Med. Chem.*, **26**(1991)291.
7. G. Rima, J. Satgé, H. Sentenac-Roumanou, M. Fatome, C. Lion and J. D. Laval, *Eur. J. Med. Chem.*, **28**(1993)761.
8. G. Rima, J. Satgé, H. Sentenac-Roumanou, M. Fatome, J. D. Laval, C. Lion, O. Alazard and P.Chabertier, *Appl. Organomet. Chem.*, **8**(1994)481.
9. G. Rima, J. Satgé, H. Sentenac-Roumanou, M. Fatome, J. D. Laval, C. Lion and R. Dagiral, *Appl. Organomet. Chem.*, **10**(1996)113.
10. G. Rima, J. Satgé, H. Sentenac-Roumanou, M. Fatome, J. D. Laval, C. Lion, C. Thiriot, R. Dagiral and C. Martin, *Main Group Met. Chem.*, **20(4)**(1997)255.
11. F. I. Carroll, H. M. Dickson and M. E. Wall, *J. Org. Chem.*, **30**(1965)33.
12. G. Dousse, J. Satgé and M. Rivière-Baudet, *Synth. React. Inorg. Met.-Org. Chem.*, **3**(1973)11.
13. M. Lesbre, P. Mazerolles and J. Satgé in: *The Organic Compounds of Germanium*, John Wiley and Sons, New York, (1973).
14. J. Satgé, M. Lesbre and M. Baudet, *C. R. Acad. Sci. Paris. Ser. C.* **259**(1964)4733.
15. J. Satgé and M. Baudet, *C. R. Acad. Sci. Paris. Ser. C.* **263**(1966)435.
16. J. Corbin, K. F. Miller, N. Pariyadath, S. Wherland, A. E. Bruce and E. I. Stiefel, *Inorg. Chim. Acta*, **90**(1984)41.
17. J. Satgé, Propriétés et applications biologiques de dérivés organométalliques du silicium, du germanium, et de l'étain, rapport de mise au point A. E. P. A., juin 1981.
18. G. Atassi, *Rev. Silicon Germanium Tin Lead Compd.*, **8**(1985)219.
19. J. S. Thayer, *Rev. Silicon Germanium Tin Lead Compd.*, **8**(1985)133; *Appl. Organomet. Chem.*, **1**(1987)227.
20. T. K. Gar and V. F. Mironov, in: *Review of the Biological Activity of Germanium Compounds*, Niitekhim, (1982), Moscow.
21. E. Lukevics and L. Ignatovich, *Appl. Organomet. Chem.*, **6**(1992)113.
22. E. Lukevics, L. Ignatovich, N. Shilina and S. Germane, *Appl. Organomet. Chem.*, **6**(1992)261.
23. E. Lukevics, S. Germane and L. Ignatovich, *Appl. Organomet. Chem.*, **6**(1992)543.
24. F. Anger, J. P. Anger, L. Guillou and A. Papillon, *Appl. Organomet. Chem.*, **6**(1992)267.
25. R. Tacke, D. Reichel, P. G. Jones, X. Hou, M. Waelbroeck, J. Gross, E. Mutschler and G. Lambrecht, *J. Organomet. Chem.*, **521**(1996)305.

**Received: April 14, 1998 - Accepted: April 24, 1998 - Received in revised camera-ready format: April 28, 1998**

